# Cost of goods sold analysis and recommendations to reduce costs of co-packaged mifepristone–misoprostol for medical abortion

**DOI:** 10.1186/s12978-020-01012-8

**Published:** 2020-11-04

**Authors:** Lester Chinery, Chadia Allaouidine, Alessandra Tomazzini, Melanie Larson, A. Metin Gülmezoglu

**Affiliations:** Concept Foundation, Avenue de Sécheron 15, 1202 Geneva, Switzerland

## Abstract

**Objective:**

Understanding the price components of the mifepristone/misoprostol (combi-pack) for medical abortion to improve access is critical for identifying strategies to reduce product costs for quality-assured formulations and expanding its availability and use.

**Methods:**

We constructed a cost of goods sold analysis using data collected from manufacturing companies in Bangladesh, China and India supported by publicly available information related to the product formulation, active pharmaceutical ingredients (API), manufacturing location, manufacturer profiles and other individual model components. Key model components were the active pharmaceutical ingredients (quality-assured or not), excipients, labour cost, operating cost and packaging.

**Results:**

Combi-pack direct production cost ranges from US$1.08 for finished products which are not quality assured to US$3.05 for products containing quality assured active pharmaceutical ingredients, which means that with a 30% administrative fee applied to those prices, it could be made available between US$1.40 and US$3.97 depending on location, manufacturer’s profile, optimal market situation and the quality of the active pharmaceutical ingredients. The main model component impacting on the cost range is the purchase price of mifepristone active pharmaceutical ingredient and the current differential between quality-assured material supported by adequate documentation and API for which quality assurance cannot be demonstrated. Compared to India cost of goods sold is lower in Bangladesh primarily due to lower operating costs, including the cost of labour.

**Conclusions:**

It is feasible to lower the cost of quality-assured combi-packs, through reducing mifepristone API cost and selection of the manufacturing location. However, manufacturers need to be incentivised to achieve WHO pre-qualification with a carefully built business case and require support in identifying and sourcing competitively priced material and manufacturing products to the necessary standard.

## Plain English summary

We conducted a study to assess the price components of two medicines used together for medical abortion. Identifying the individual price components is helpful to understand whether product costs can be reduced and access increased, to quality-assured products. We compared production costs for manufacturing in Bangladesh and India, two countries with substantial generic manufacturing capability and, we also looked at price differentials for manufacturing a quality-assured or non-quality-assured product. Quality-assured products are those approved by stringent regulatory authorities or achieved World Health Organization Pre-Qualification status. We found that mifepristone–misoprostol combi-packs could be made available between US$ 1.40 and US$ 3.97 depending on location, manufacturer’s profile, optimal market situation and the quality of the active pharmaceutical ingredients. The purchase price of mifepristone active pharmaceutical ingredient is the main model component impacting on the cost range. Compared to India cost of goods sold is lower in Bangladesh primarily due to lower operating costs, including the cost of labour. It is feasible to lower the cost of quality-assured combi-packs, through reducing mifepristone active ingredient cost and selection of the manufacturing location.

## Introduction

Access to quality-assured (QA) medicines (defined as products that have been WHO prequalified or approved by a stringent regulatory authority https://www.who.int/medicines/regulation/sras/en/) for the termination of pregnancy remains limited for women in need in many parts of the world. One of the key barriers limiting procurement and access to these essential medicines is affordability in low-resource settings. Understanding the price components of the combination mifepristone/misoprostol (combi-pack) for medical abortion and determining if this may be impacting access is critical for identifying strategies to reduce product costs and expanding its availability and use.

WHO guidelines for medical abortion recommend the use of 1 tablet of mifepristone 200 mg and 4 tablets of misoprostol 0.2 mg, and a combi-pack containing both products in the one packaging has been developed as the optimal product for this indication.

The price of a combi-pack containing one tablet of mifepristone and four tablets of misoprostol is currently marked by significant variances. Based on prior work undertaken by Concept Foundation, International Planned Parenthood Federation and Gynuity Health Projects, the cost of medical abortion commodities was found to vary significantly across brands and products (i.e., misoprostol, mifepristone and the combi-pack) as well as geography in low- and middle-income countries (LMIC) [1]. Therefore, it is difficult for procurement institutions and other customers to evaluate whether the prices offered by manufacturers are reasonable, accurately reflecting true production costs, whether they could be lowered, or if low cost reflects a lack of documented quality-assurance.

Customer price is driven by a range of components which can be grouped under production and commercial. Commercial cost factors may vary drastically from one manufacturer to another and involve different considerations such as a manufacturer’s business positioning, assessment of the competition, investments, demand, commercial risks, size and scope of operation and internal cash flow situation or business objective.

**Fig. 1:**
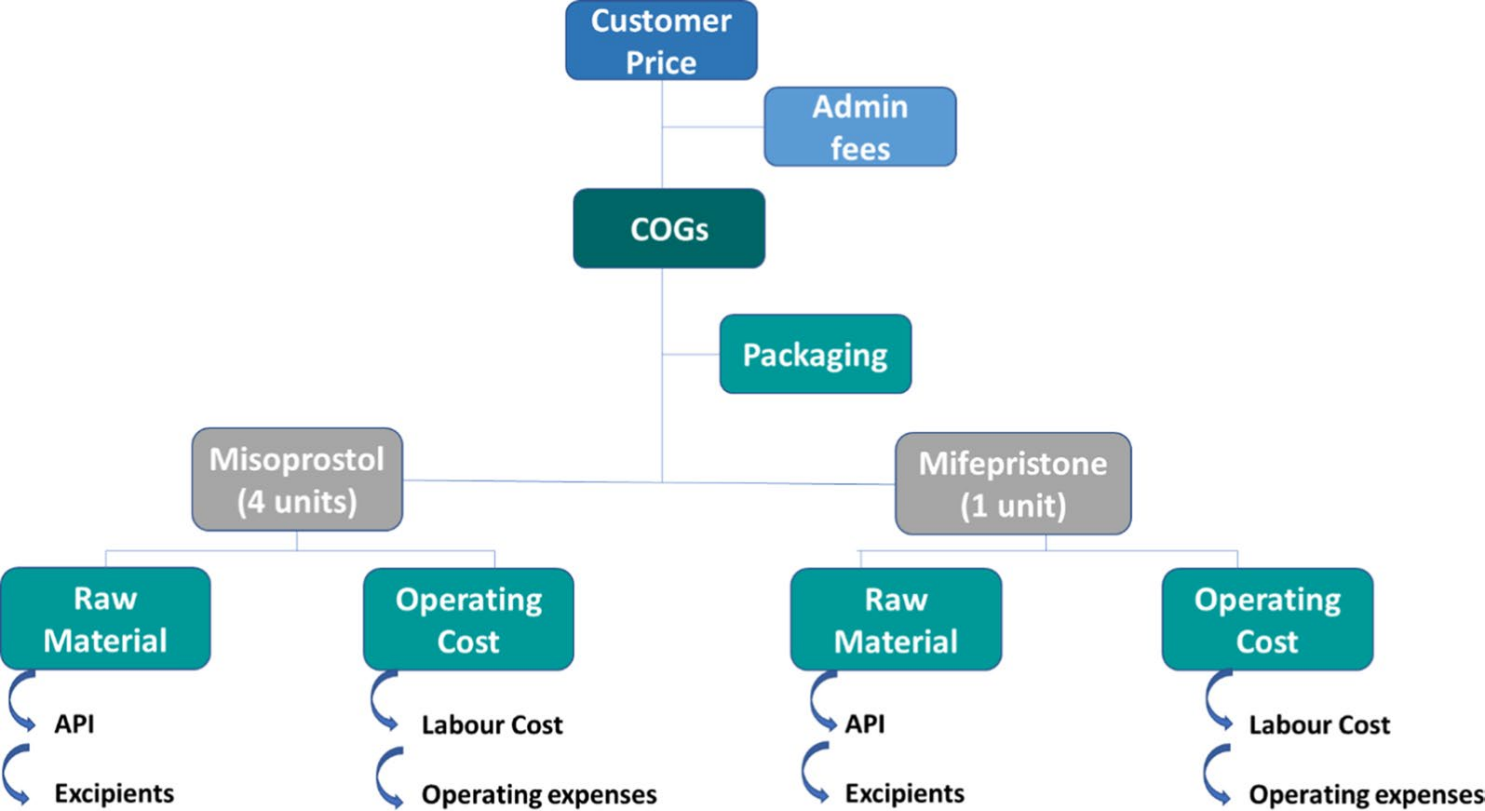
COGS model components

Production cost factors, however, are similar across manufacturers. Production costs, which will be referred to as the cost of good of sold (COGS), can be defined as the sum of the direct costs attributable to the production of the goods sold by a company. It includes the cost of the materials used in creating the product (i.e., active pharmaceutical ingredient (API), excipients and packaging), operating expenses (i.e., utilities maintenance, electricity and energy costs), and the direct labour costs used to produce the goods (Fig. 1). The COGS excludes administrative fees—distribution costs and sales force costs (https://www.investopedia.com/terms/c/COGS.asp) and does not include the profit margin.

To elucidate some of the key costs at the point of manufacturing that drive production costs upstream, a COGS analysis was performed. The objective of this report is to provide insights into the COGS of the combi-pack, in order to identify if the current costs of quality assured brands are reasonable and assess if, and if so how, the costs could be reduced through targeted initiatives.

## Methods

To build a model applicable to a broad range of combi-pack manufacturers, several assumptions related to the product formulation, manufacturing location, manufacturer profiles and the COGS model components were made.

### Product formulation

The combi-pack is the combination of two drugs: misoprostol (4 tablets) and mifepristone (1 tablet) in the strength 0.2 mg and 200 mg respectively, packaged and ideally blistered in Alu/Alu (Table 1).

**Table 1:** Misoprostol and mifepristone formulations

Misoprostol	Measurement^a^	Per tablet	Per combi-pack
Misoprostol solid dispersion (1% in HPMC)	mg	20.20	80.80
Microcrystalline cellulose	mg	172.10	688.50
Sodium starch glycolate	mg	6.30	25.20
Hydrogenated castor oil	mg	1.40	5.60
Mifepristone			
Mifepristone	mg	200.00	200.00
Magnesium stearate	mg	3.75	3.75
Corn starch	mg	75.75	75.75
Microcrystalline cellulose	mg	60.00	60.00
Povidone	mg	14.50	14.50
Silica colloidal anhydrous	mg	6.75	6.75

^a^ The API procured for most part of the manufacturers is a dispersion of 1% misoprostol in hydroxypropyl methyl cellulose (HPMC). For this reason, the weight of the API introduced into the process is proportionally adjusted to ensure that each tablet will contain 0.2 mg of Misoprostol. The weight of the misoprostol component (1% misoprostol in HPMC) is therefore 20.2 mg per tablet, however it contains 0.2 mg of misoprostol

### Manufacturing location

Guided by the objective to increase access globally and particularly in high-need, low-resource settings, the COGS model explores the production cost of manufacturers located in India and Bangladesh. These countries were selected for the following reasons:

- Both are/expected to continue to lead the pharmaceutical market in terms of combi-pack supplies for LMIC.
- Due to differences in the economic development progress between the two countries [2], the model can explore different production cost configurations.
- Concept Foundation has longstanding experience in collaborating with manufacturers located in these markets and consequently, access to the required data.

### Manufacturer profiles

The model assumes that the manufacturers are already producing the combi-pack with the following implications:

- A discount of 15% is applied to the base cost of the raw materials to reflect the existing commercial relationship between the manufacturer and its suppliers.
- The model does not assume a significant upfront investment, which needs to be recovered in a short period of time as may be the case for a new manufacturing market entrant.

For comparison purposes, the model includes the following additional assumptions:

- The raw material, API included, is not manufactured internally and needs to be purchased from a third-party;
- 97% (Based on United States Pharmacopeia (USP) Grade) assay for the correction of mifepristone API and 95% (Based on WHO Pharmacopeia) assay for the correction of the misoprostol API. Usually manufacturers adjust the amount of API weight/added to the batch based on the assay results and/or on anhydrous basis to obtain theoretically 100% of the amount of each ingredient in compounded formulations. Calculations must account for the active ingredient, or active moiety, and water content of drug substances, which includes that in the chemical formulas of hydrates;
- 25,000 tablets of misoprostol compressed per hour;
- The depreciation on equipment is set at 15%/year;
- The batch size is set at 100,000 units for each product; which in terms of combi-pack is equivalent to:
  - 100,000 units of mifepristone;
  - 400,000 units of misoprostol;
- All units that are manufactured can be commercialised (During the production process the manufacture may experience a loss of tablets which is commonly referred as the yield. The global yield is defined by losses of powder (all components mixed) at compounding, losses of tablets during compression, blisters during secondary packaging, etc. which could be caused by human failure, low quality or variability on raw materials, losses during the transferences (retention on vacuum systems, etc.), old fashion/obsolete machinery. The model didn’t explore the impact of the global yield as part of the manufacturing process).
- Operating expenses.
  - Equipment is manufactured and insured in accordance with European Union standard practices.
  - Manufacturing is assumed to operate in compliance with internationally accepted GMP (if a manufacturer is not GMP compliant then their operating costs may be lower).
  - The manufacturer doesn’t profit from any condition entreprise zone with particular tax benefits for instance.

### The COGS model components

The COGS model includes five components:

1. API (QA product vs non-QA product);
2. Excipients;
3. Labour cost (manufacturing; support area workers; quality unit);
4. Operating cost (infrastructure maintenance, depreciations);
5. Packaging (Alu/Alu; other packaging (leaflet, carton, label, etc.)).

While not part of the standard definition of the COGS model, administrative fees is an important consideration to understand if the final buyer/customer product price is reasonable. Consequently, we explored administrative fees as part of the final cost calculation.

Administrative fees usually cover indirect coordination and running costs such as commercialization activities, which are not directly attributable in the production cost breakdown. However, this indicator tends to consider fewer tangible elements such as cost recovery relating to regulatory activities and upfront research and development investments, which, once recovered, are not subsequently deducted from the final product price. As a result, a level of profit is often hidden behind this indicator, which can explain the lack of transparency over this component of the price on the part of manufacturing companies. Based on our experience in COGS modelling and working with multiple manufacturers, the administrative fee is estimated at between 30 and 40% of the direct production cost per unit. Manufacturers may add a further profit margin, in addition to the administrative fee, dpending upon the range of elements already included and other external market factors.

In summary, key production components are important to understanding the final price of the COGS, and the admistrative fee is critical to identifying at which price point it may start to be viable and interesting for a manufacturer to produce a drug.

The COGS model was based on our knowledge and work with manufacturers and on information collected from pharmaceutical manufacturers and chemical suppliers (Pharmacompass: www.pharmacompass.com; Seair Exim Solutions: https://www.seair.co.in; Spectrum Chemical: https://www.spectrumchemical.com).

### Parameters investigated to explain the price variances of the combi-pack

To identify the drivers behind combi-pack costs we explored each of the different components in-depth. Based on these parameters, two manufacturer profiles were developed and explored in two different manufacturing locations.

- Profile A: A company which does *NOT procure* QA API to manufacture the combi-pack (it can also be assumed that such companies may also not be manufacturing in compliance with international GMP standards as there is evidence that in both markets products are available below these price points).
- Profile B: A Company which *procures* QA API to manufacture the combi-pack.

### Limitations of the model

A COGS model cannot exactly reflect the specificity of each company and the circumstances. Therefore, the model must be based on selected parameters applicable across manufacturers. As a result, the following elements were not assessed as part of the process:

- *Import taxes on raw material*. A tax should be applied to the raw material imported towards the production of the combi-pack. However, some manufacturers can procure the raw material in their local market. For comparison purposes, this element was removed from the model.
- *Raw material self-production*. The model assumes that the manufacturer must purchase all raw material components of the Finished Pharmaceutical Product (FPP). However, in some cases, the manufacturer is also the producer of the API composing the FPP. For comparison purposes, this element was removed from the model.
- *Packaging*. The combi-pack is currently sold packaged in different material such as Alu/Alu or plastic (e.g. Alu-PVC, Alu-PVdC. OPA/Alu/PVC). Guided by the objective of assessing the costs pertaining to QA combi-pack, the model didn’t explore the impact of the different packaging materials on COGS and the costing is based upon the use of single Alu/Alu blister packs containing both the mifepristone and misoprostol tablets. WHO prequalified combi-packs are exclusively packaged in single Alu/Alu blisters. Other packaging such of PVC or PVdC were identified as material which could affect the stability of the product adversely.

## Results

The COGS modelling exercise showed that the combi-pack direct production cost ranges from US$1.08 to US$3.05 (Total COGS), which means that with a 30% administrative fee applied to those prices, a combi-pack could be made available at a cost of between US$1.40 and US$3.97 depending on location, manufacturer’s profile and optimal market situation (i.e. all manufactured units are consumed).

### Results for a combi-pack manufactured in India

The key variable determining the COGS for product manufactured in India is the cost of mifepristone API. Using QA API at a (discounted) cost of US$7036/kg results in a cost per unit of $3.97 as demonstrated under Profile B below. Using API that is not demonstrably QA and available on the market the cost per unit is $2.09 as shown under Profile A. The cost of API for this profile is US$875/kg (which is not the lowest price point available, but which reflects current purchasing prices based upon limited evidence from manufacturers). The price outcomes for Profile B is broadly consistent with existing LMIC public sector access pricing from Sun Pharmaceuticals for the Medabon combi-pack of between US$3.75 and US$4.00.

### Results for a combi-pack manufactured in Bangladesh

The key variable determining the COGS for product manufactured in Bangladesh is the cost of mifepristone API. Using QA API at a (discounted) cost of US$7036/kg results in a cost per unit of US$3.29 as demonstrated under Profile B below. Using API that is not demonstrably quality assured the cost per unit is US$1.40 as shown under Profile A. The cost of API for this profile is US$875/kg (which is not the lowest price point available, but which reflects current purchasing prices based upon limited evidence from manufacturers). There are currently no QA combi-packs manufactured in Bangladesh.

### India v Bangladesh

With both models containing identical direct costs, the key cost differentials between India and Bangladesh relate to the operating and maintenance costs and cost of labour, both directly for the purpose of manufacturing and indirectly with labour as a component of the operating and maintenance costs line item. The result of this, is that it may be feasible to achieve a price point of $3.29 for a QA combi-pack if it were manufactured in Bangladesh. Furthermore, if there was greater competition and/or quantifiable market incentive, resulting in a reduction of the cost for quality assured and documented mifepristone API, combi-pack unit prices in the future could be even lower.

In practice, there will be a wide variety of variables, unique to individual companies which may increase or decrease operating costs. This paper has established thoroughly researched and accurate benchmarks for making comparison in support of the central premise.

## Discussion

Considering the WHO, UNFPA and Concept Foundation commitment to the availability of QA products, we believe this COGS analysis will support further thinking and work towards access to affordably priced combi-packs in LMIC. We regard Profile B i.e. QA profile as the standard/existing profile for further analysis.

Based upon India production costs, the COGS is estimated at US$3.05 and with the addition of administrative fees, the commercial offer price to institutional public sector and social marketing organizations (SMO) customers is estimated at US$3.97. For Bangladesh, the COGS is estimated (considering lower labour and operating costs) at US$2.53 for the COGS and with the addition of administrative fees, the commercial offer price to institutional public sector and SMO customers is estimated at US$3.29.

The COGS model details for Profile B, showed that the two principal drivers of the cost fluctuation identified are, procurement of QA API mifepristone and the operating expenses (driven by the individual countries cost of living reflected in operational costs and the cost of labour and other linked costs) (Fig. 1).

### Procurement of QA API mifepristone

Procurement of QA and documented mifepristone API accounts for the largest single expense in the combi-pack COGS, accounting for 48% of the combi-pack production cost in the Indian setting and 57% of the combi-pack production cost in Bangladesh. Through confidential discussions with manufacturers, it was possible to identify an indicative purchase price of quality assured mifepristone API at US$8278 per kg, while non-QA mifepristone API can be sourced at an average price of only US$875 per kg (and less). The ratio between non-QA to QA API mifepristone cost is 1:9.4. For comparison, the ratio for non-QA to QA misoprostol API is 1:3.4 (QA API at US $2941/kg). As a result, using QA mifepristone API increases the product cost to US$1.30/tablet while the purchase of QA misoprostol API only increases the product cost by US$0.04/tablet (Total of US$1.45 difference between COGS for the QA combi-pack vs Non-QA combi-pack).

The pie charts below highlight the increasing impact on the cost configuration depending on the quality of the product. While QA mifepristone API accounts for 95% of the costs of raw material, this reduces to 78% for non-QA API.

**Fig. 2:**
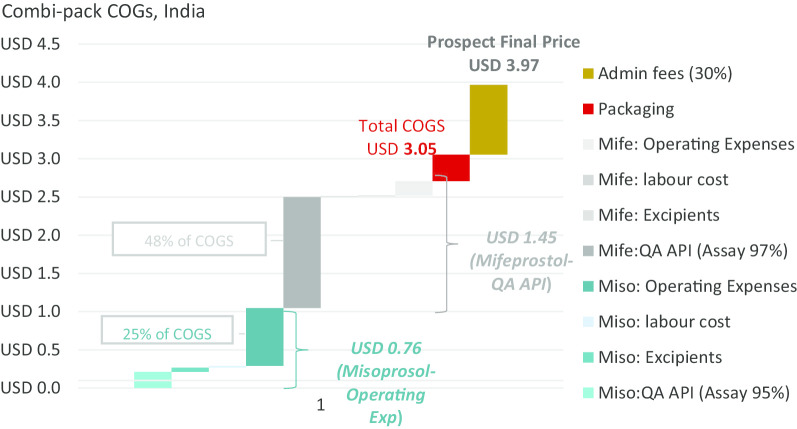
COGS elements in India

**Fig. 3:**
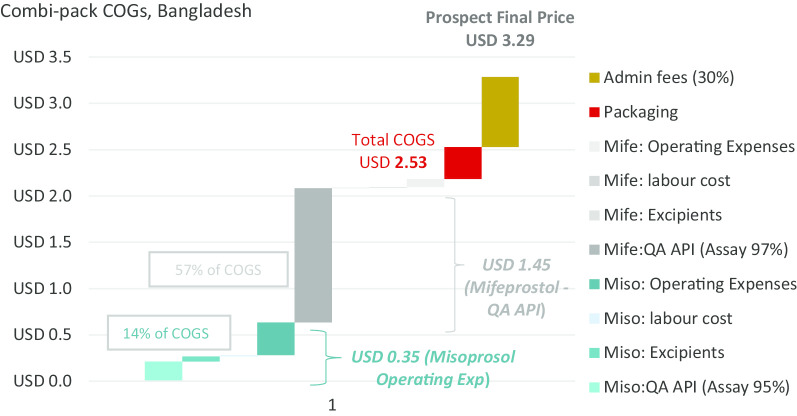
COGS elements in Bangladesh

The COGS elements graphs (Figs. 2 and 3) show that operating expenses relating to misoprostol are the second highest expense and account for US$0.76 of the Indian combi-pack costs while only US$0.35 for the model developed for Bangladesh. This indicator includes utilities maintenance such as cleaning or electricity. It is therefore a heterogenous factor—composed of indirect labour costs but also energy costs for example.

The COGS model assumes that the manufacturer uses EU equipment subject to high maintenance standard practices and insured adequately. The model doesn’t explore other factors pertaining to operating expenses such as equipment origin, maintenance procedures, insurance setting or enterprise zones options which could decrease operating expenses.

When comparing the COGS of India to Bangladesh, we see that under similar scenarios, the costs of the combi-pack are systematically lower in Bangladesh than in India. The COGS model shows that the explanation for this outcome is not primarily due to cheaper direct labour costs in Bangladesh but rather the lower overall operating expense costs in the country, of which labour is both a direct and indirect component.

Despite the difference in direct manufacturing labour costs between the countries, because of the importance of the batch size, the impact of labour costs on the total cost per tablet is marginal, as illustrated in the charts below. Moreover, the model indicates that the competitive advantage in terms of production cost of Bangladesh over India is instead driven by indirect operating expenses. This can be observed through analysing the operating expenses relating to the misoprostol tablet. The operating expenses account for 56% of the production costs of one tablet of misoprostol in Bangladesh vs. 72% for one tablet of misoprostol in India.

Overall, this analysis is in alignment with general macro-economic theory which highlights the challenge for more advanced economies to remain competitive in the manufacturing of cheap goods. Therefore, due to its economic dynamics and growth trajectory, Bangladeshi manufacturers are expected to be better placed to offer a lower priced QA combi-pack product for the medium term.

### Can the combi-pack price be lowered?

The COGS of the combi-pack is driven by both macro- and micro economic factors. The macro level components, which impact operating expenses, are related to economic indicators of sovereign states which will only change as a result of progress (or otherwise) and changes to the broader national context which determine energy costs and labour for example. This may include providing manufacturers with tax breaks or other incentives. However, at the micro level, we have identified that the most effective way to reduce cost, whilst maintaining quality is through a decrease in the QA mifepristone API price, which is the most significant cost factor in the model. This could be achieved by creating competition among suppliers of API for QA material, which requires either an incentive to encourage investment and lower per kg price points, or a much larger market for quality assured combi-packs—and probably both.

### Case scenario: impact of reduced cost of API mifepristone on combi-pack COGS

The modelling undertaken and shown above is based upon a price of US$8278/kg with a discount of 15% making the material available at US$7036/kg. If mifepristone API was made available to manufacturers at US$5000/kg, a price which we believe is both feasible and viable for the producers, the reduction by itself, would be sufficient to reduce the cost of the combi-pack. The tables below provide an indication of how this would impact the existing COGS model for manufacturing in both India and Bangladesh (Tables 2, 3, 4 and 5).

**Table 2:**
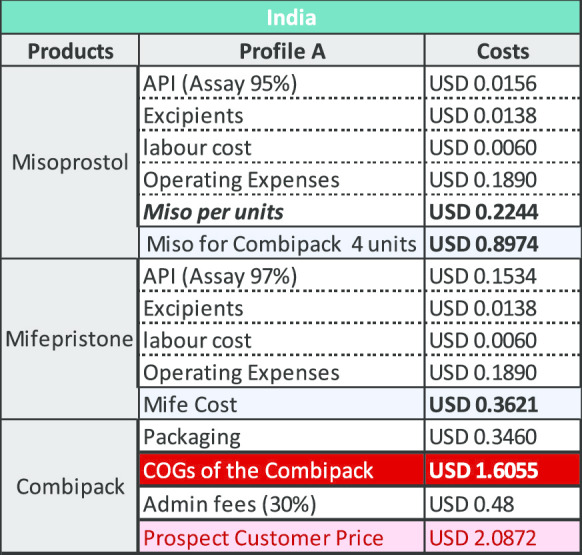
COGS for company with Profile A in India

**Table 3:**
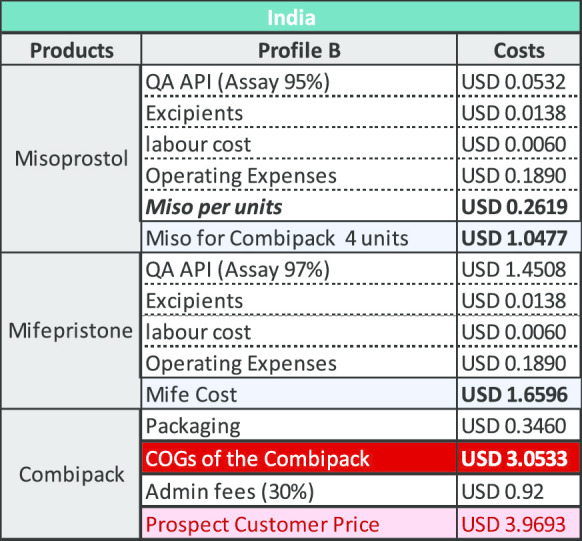
COGS for company with Profile B in India

**Table 4:**
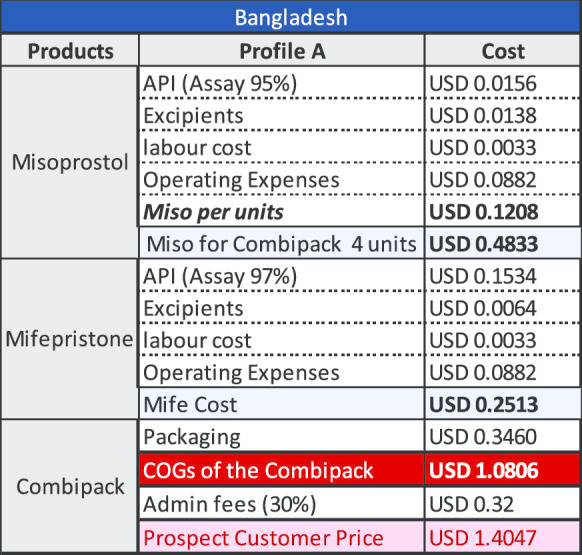
COGS for company with Profile A in Bangladesh

**Table 5:**
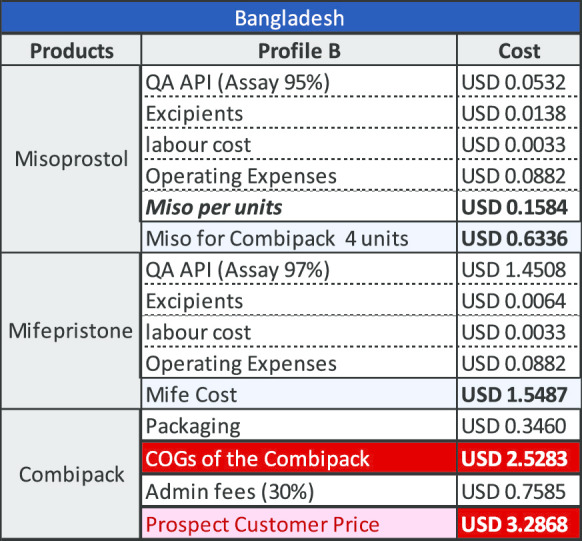
COGS for company with Profile B in Bangladesh

The low cost of non-QA mifepristone API gives an indication that this high price cannot solely be explained by production cost considerations. Consequently, trying to implement reforms on the market dynamics may be the most optimal pathway. This can be implemented in a couple of ways with market shaping interventions such as volume guarantees to drive the current supplier(s) to decrease their price or more liberal initiatives supporting greater competition through additional QA mifepristone API and consequently greater competition and choice of combi-packs in markets.

**Fig. 4:**
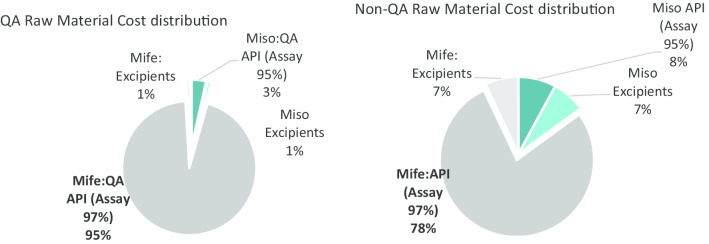
Cost distribution of raw materials

**Fig. 5:**
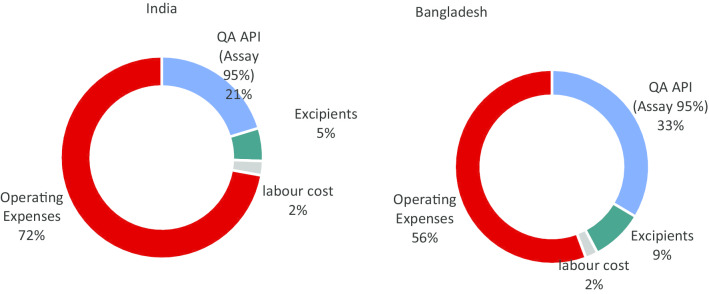
Misoprostol tablet production cost distribution

A decrease based on the US$5000/kg example above on the cost of QA mifepristone API would have positive impact for both manufacturers located in India and Bangladesh. An Indian manufacturer could potentially offer a decreased combi-pack price of US$3.42 and in Bangladesh, the price could reduce to US$ 2.74 as shown in the Profile C tables below. Greater reductions in the cost of QA mifepristone API would accordingly, reduce these prices even further (Figs. 4, 5).

## Limitations

The paper is based upon the existing market situation and considers price reduction opportunities in the context of reducing the cost of API, labour and operating expenses through increasing competition (API) and location of manufacturing. There are conceivably other additional scenarios for achieving a similar level of price reduction, for example, an external intervention by donor agencies whereby a level of subsidy is provided to incentivize companies to improve product quality and lower prices in return for direct financial support and/or through guaranteeing volumes for a time-limited period in an effort to shape a larger future market.

The impact of the packaging material was not explored in-depth as part of the COGS model. However, this is recognized to play a role as a cost component of the COGS. The use of Alu/Alu material by manufacturers was estimated at US$0.346 to produce misoprostol in Bangladesh. At the present time, manufacturers in Bangladesh do not co-blister misoprostol and mifepristone and the cost of doing so as well as the cost of Alu/Alu packaging associated with the QA combi-pack may also contribute to disincentivizing manufacturers located in Bangladesh to enter its products into the QA market.

## Conclusions

Considering that the final price relies heavily on an individual manufacturer’s commercial interests, it is important to ensure a business case exists or is developed for QA mifepristone API manufacturers to sustainably produce the material at a lower price (Tables 6 and 7).

**Table 6:**
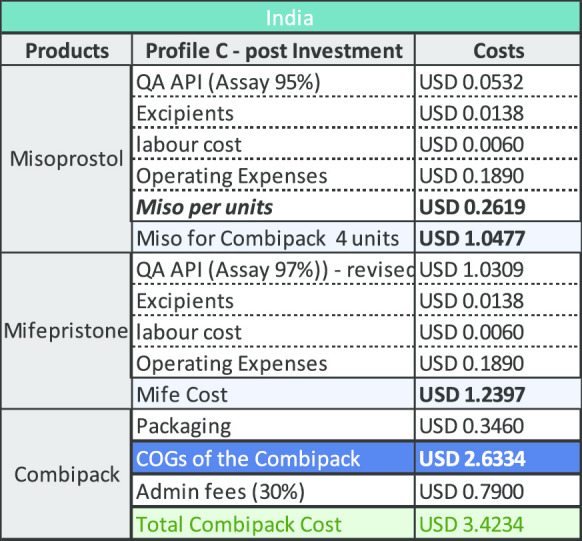
Impact of lower priced QA mifepristone API on costs in India

**Table 7:**
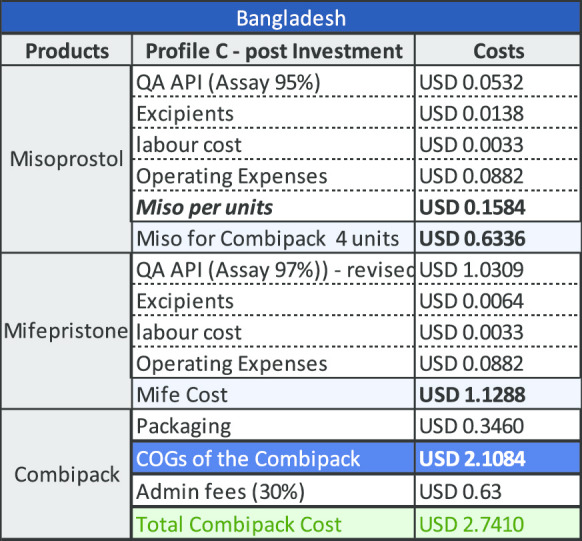
Impact of lower priced QA mifepristone API on costs in Bangladesh

We recommend conducting market assessment activities on the combi-pack market and invest in advocacy activities to establish and identify market opportunities for QA mifepristone API manufacturers and partner with finished product manufacturers of combi-packs.

Based upon our market assessment activities, ensuring that buyers/customers prioritise quality over price considerations remains the key commercial challenge that manufacturers must consider before entering into the QA market for combi-packs. However, additional API manufacturers can be incentivised by technical support provided to achieve WHO prequalification especially in Bangladesh.

## Data Availability

The contact author can be contacted for publicly available data access. Some details are proprietary information of the manufacturers and not available.

## Abbreviations

API: Active pharmaceutical ingredient
COGS: Cost of goods sold
LMIC: Low and Middle Income Countries
QA: Quality-assured
USP: United States Pharmacopeia
WHO: World Health Organization
